# Association of sleep duration with apolipoproteins and the apolipoprotein B/A1 ratio: the China health and nutrition survey

**DOI:** 10.1186/s12986-017-0237-8

**Published:** 2018-01-05

**Authors:** Huihui Ren, Zhelong Liu, Xinrong Zhou, Gang Yuan

**Affiliations:** Department of Internal Medicine, Tongji Hospital, Huazhong University of Science and Technology, Wuhan, Hubei 430030 People’s Republic of China

**Keywords:** Apolipoprotein A1, Apolipoprotein B, Apolipoprotein B/Apolipoprotein A1 ratio, Sleep duration

## Abstract

**Background:**

Short sleep duration has been related to established cardiovascular risk factors, likely obesity, diabetes, hypertension and dyslipidaemia. However, to the best of our knowledge, the associations between sleep duration and apolipoprotein concentrations and their ratios have not been investigated to date. This study aimed to explore the independent relationship of sleep duration with apolipoprotein (apo) A1, apoB and the apoB/apoA1 ratio in a Chinese adult population.

**Methods:**

Data from 7381 participants, aged 18 to 75 years, from the National Health and Nutrition Survey 2009 were analysed in this cross-sectional study. Participants were divided into 3 categories according to sleep duration: ≤6, 7–8, and ≥9 h. Logistic regression analysis with odds ratios was employed to assess the association between sleep duration and apo profile.

**Results:**

Using 7–8 h of sleep as a reference, short sleep duration was associated with significantly increased odds of elevated apoB (OR =1.75, 95% CI 1.12–2.72), whereas long sleep duration was correlated with a decreased (but not statistically significant) risk for elevated apoB (OR =0.86, 95% CI 0.54–1.38) among females after controlling for covariates. Among males, long sleep duration was only marginally related to decreased odds ratios for elevated apoB/apoA1 ratio after adjustment for covariates (OR =0.78, 95% CI 0.6–0.99).

**Conclusions:**

These results indicate that short sleep duration is strongly associated with an increased risk of elevated apoB levels in women and that long sleep duration is correlated with decreased apoB/apoA1 levels in men. Sleep hygiene management could serve to treat and prevent cardiovascular diseases by altering unfavourable apo profile.

## Background

Apolipoproteins and the apolipoprotein B/apolipoprotein A1 (apoB/apoA1) ratio have been reported to be independent predictors of risk of cardiovascular events. A meta-analysis of 23 prospective studies demonstrated that low apoA1 levels and high apoB and apoB/A1 levels were associated with an increased risk of coronary heart disease (CHD) [1]. Findings from previous research showed that apoB and apoA1 levels were superior to total cholesterol and low-density lipoprotein cholesterol (LDL-C) in predicting the future risk of fatal myocardial infarction [2]. Epidemiological studies have also demonstrated that apolipoproteins and the apoB/apoA1 ratio represent potential risk factors for the development of type 2 diabetes mellitus (T2DM) [3], insulin resistance [4], non-alcoholic fatty liver disease [5], metabolic syndrome [6], and cancer [7]. Multiple adverse health outcomes have been linked to low apoA1 levels, high apoB levels and high apoB/apoA1 ratios, leading to interest in developing strategies to manage abnormal apo variables for the prevention or treatment of associated metabolic and cardiovascular diseases.

Sleep is a behavioural risk factor that has been considered an additional factor adversely affecting multiple public health concerns. Although there are several inconsistencies in the published literature [8, 9], large-scale studies have reported that both short and long sleep durations are independently associated with an increased risk of diabetes [10], hypertension [11], cardiovascular diseases [12], and all-cause mortality [13]. These observations supported the “U-shaped” association between sleep duration and increased risk of adverse health outcomes. There is also evidence that sleep duration is linked to atherosclerotic dyslipidaemia. For example, epidemiological studies have demonstrated that short sleep durations were significantly associated with increased total cholesterol, LDL-C levels and low high-density lipoprotein cholesterol (HDL-C) levels [14, 15]. However, studies exploring the association of sleep duration with apolipoproteins and the apoB/apoA1 ratio are scarce. It is well-known that apolipoproteins are closely linked to serum lipid concentrations and are responsible for the synthesis, metabolism, as well as structural stability of lipoprotein particles, which contain triglycerides, free cholesterol, cholesterol esters, and apolipoproteins [16]. Additionally, findings of an epidemiological study have indicated that modifiable lifestyle factors could strongly influence apo concentrations [17]. Therefore, apo variables were hypothesized to correlate with sleep duration, as well.

Since sleep duration was an established modifiable lifestyle factor, understanding the correlation between sleep duration and apo variables could be of great importance to develop behavioural strategies to prevent the cardiometabolic events and mortality associated with unfavourable apo profile. To explore the relationship between sleep duration and the levels of apoA1, apoB and the apoB/apoA1 ratio, we analysed the nationally representative data from the 2009 China Health and Nutrition Survey (CHNS).

## Methods

### China health and nutrition survey

The CHNS is a longitudinal cohort survey that is conducted to examine the health and nutritional status of its population. The survey began in 1989, with a total of nine waves (1989, 1991, 1993, 1997, 2000, 2004, 2006, 2009, 2011) using a multistage random-cluster process to select samples surveyed from nine provinces. The provinces varied largely in geography, economic development public resources, and health indicators covering Heilongjiang, Liaoning, Shandong, Jiangsu, Henan, Hubei, Hunan, Guangxi, and Guizhou. Counties in each province were stratified based on income levels (low, middle, and high), and a weighted sampling scheme was used to randomly select four counties. Details of the CHNS are described elsewhere [18]. Informed consent was obtained from each participant, and the survey was approved by the institutional review committees of the University of North Carolina at Chapel Hill, the National Institute of Nutrition and Food Safety, the Chinese Centre for Disease Control and Prevention, and the China-Japan Friendship Hospital, Ministry of Health.

### Study participants

Since blood samples were first collected in 2009, we only analysed the data from the 2009 CHNS. Thus, 8102 participants aged 18 to 75 years with biomarkers were eligible for recruitment. Among these adults, individuals with pregnancy (*n* = 62), chronic renal disease (*n* = 6, estimated glomerular filtration rate (eGFR) <15 ml/min/1.73 m^2^) [19], or missing data for apoA1, apoB, the apoB/A1 ratio (*n* = 19) or sleep duration (*n* = 368) were excluded. We also excluded participants with missing values for other measures: total cholesterol, triglycerides, HDL-C, LDL-C, haemoglobin A1c (HbA1c), fast plasma glucose (FPG), hypersensitive C-reactive protein (hs-CRP), uric acid, body mass index (BMI), systolic and diastolic blood pressure, smoking status, drinking status, or obtained educational level. The remaining 7381 participants (3463 men, 3918 women) were included in our analytic sample.

### Assessment of sleep duration

Sleep duration was assessed with self-reported questionnaire. The questionnaire on physical activity included the question: “How many hours each day do you usually sleep, including daytime and night time? (hours).” Responses of the number of hours per day ranged from 1 to 18 h. Given the U-shaped relationship of sleep duration with cardiometabolic disease events [11, 12, 20], sleep duration was categorized as ≤6 (short), 7 to 8 (optimal), and ≥9 h (long) per day.

### Interviews and measurements

Sociodemographic information of the subjects, including age, sex, rural/urban sites and education level, was investigated using a self-reported questionnaire. Participants were also asked to report smoking status (never, former and current smokers), drinking habits, and self-reported medical history of hypertension, diabetes, and the use of antihypertensive drugs methods.

We measured height, weight, waist circumference (WC) and blood pressure under standardized conditions. Height was measured to the nearest 0.1 cm with participants wearing light clothing and without shoes using a portable SECA stadiometer (SECA; Hamburg, Germany). Weight was measured to the nearest 0.1 kg without shoes and in light clothing using a calibrated beam scale. The WC was measured with participants in an upright position at a navel level at the end of expiration using a tape. BMI was computed as body weight (kg) divided by height squared in metres (m^2^). After a 10-min rest, blood pressure was measured in triplicate using mercury sphygmomanometers with an interval of 3–5 min between the 2 measurements. The average of the three measurements was used in the data analysis.

Blood samples were collected from study participants after an 8–12 h overnight fast. All blood samples were immediately centrifuged and tested for glucose and HbA1c and stored at −86 °C for later analysis. The apoA1 and apoB were measured by immunoturbidimetric method with a biochemical autoanalyser (Hitachi 7600 automated analyser, Tokyo, Japan). Total cholesterol was analysed by the cholesterol oxidase-phenol and aminophenazone (CHOD-PAP) method, triglycerides by the glycerol-3-phosphate oxidase-phenol and aminophenazone (GPO-PAP) method, and HDL-Cl and LDL-C by the enzymatic method. The serum uric acid level was measured by an enzymatic colourimetric method, glucose by the glucose oxidase-phenol and aminophenazone (GOD-PAP) method, and HbA1c by a chromatograph procedure. Hs-CRP was quantified by the immunoturbidimetric method using a Hitachi 7600 automated analyser.

### Definitions

Abnormal apo variables were determined based on a previously published definition [21]. Serum apoA1 levels were defined as decreased if they were less than the sex-specific 15th percentile values (<0.85 g/L in men, <0.91 g/L in women). Serum apoB levels were defined as elevated when they were ≥ the sex-specific 85th percentile values (apoB, ≥1.17 g/L in men, ≥1.18 g/L in women). An elevated apoB/apoA1 ratio was defined as an apoB/apoA1 ratio ≥ the sex-specific 85th percentile values (≥1.15 g/L in men, ≥1.08 g/L in women). According to the Working Group on Obesity recommendations in China [22], participants were defined as normal (BMI of 18.5–24.0 kg/m^2^), overweight (BMI of 24.0–27.9 kg/m^2^) and obese (BMI 28.0 kg/m^2^). Central obesity was diagnosed according to the criteria recommended by the International Diabetes Federation for Asians [23], WC ≥90 cm for men and ≥80 cm for women.

### Statistical analysis

Statistical analyses were performed separately for men and women. The interquartile ranges (IQR) were used to describe continuous variables for their skewed distribution, whereas categorical variables were presented as proportions. The Wilcoxon rank-sum test was used to investigate the differences in demographic, anthropometric, and clinical characteristics for continuous variables, and the chi-square test was used for categorical variables between men and women. Across sleep duration groups, differences in demographic, anthropometric, and clinical parameters were evaluated by the Kruskal-Wallis test with Dunn’s post hoc test for continuous variables and the chi-square test for categorical variables. Bonferroni correction was used to adjust *P*-values for all paired comparisons.

For each gender, multivariate logistic regression analysis was conducted to assess the strength of the association between sleep duration and low apoA1 levels, high apoB levels and high apoB/apoA1 ratios after adjustment for multiple confounding variables, with those who sleep 7–8 h per day as the reference group. The covariate included in the first model was age. Model 2 was further adjusted for urban/rural region, education level, smoking status, alcohol use and medical history of diabetes or hypertension. Model 3 was adjusted for all the variables in model 2 plus systolic/diastolic blood pressure, FPG, HbA1c, urid acid, and hs-CRP. Model 4 was adjusted for BMI and WC in addition to the mediating variables in model 3. The collinearity between independent variables was assessed by variance inflation factor [24]. For each gender, the unadjusted ORs between the dependent variables (apoA1, apoB and the apoB/A1 ratio) and sleep duration were identified by univariate logistic regression analysis. Statistical significance was defined at a two-tailed *P* value <0.05. All statistical analyses were performed using IBM SPSS statistics version 19.0 (SPSS, Chicago, IL, USA).

## Results

Men were more likely to report smoking, drinking and diabetes, and women were more likely to report having less than a high school education (Table 1). In addition, men had higher median values for WC, systolic and diastolic blood pressure, FPG, hs-CRP, uric acid, triglycerides, and apoB/apoA1 ratio than women (*P* < 0.05), whereas women had greater total cholesterol, HDL-C, LDL-C, apoA1 and LPA levels than men (*P* < 0.05). However, BMI, apoB and sleep duration were not significantly different between sexes. Due to the significant differences in apo profile between men and women, we analysed the data by sex.

**Table 1:** Basic characteristics of the study population by sex

	Men	Women	
Characteristic	*n* = 3463	*n* = 3918	
Age (years)	50.1 (39.3–59.6)	49.9(40–59.3)	0.586
Education < high school (%)	71.3	79.2	0.0001
Rural region (%)	68.7	67.4	0.236
Current smoking (%)	56.1	3.7	0.0001
Drinking (%)	61.6	9.3	0.0001
Medical history			
Hypertension (%)	11.7	12.6	0.245
Diabetes (%)	3.4	2.2	0.003
Systolic blood pressure (mmHg)	121(113–133)	120(110–131)	0.0001
Diastolic blood pressure (mmHg)	81(75–89)	80(71–85)	0.0001
BMI (kg/m2)	23.2 (21–25.5)	23.1(21–25.6)	0.969
WC (cm)	84(77–91)	80(74–88)	0.0001
FPG (mg/dl)	92.3(84.9–102.1)	91.4(84.6–99.9)	0.001
HbA1c (%)	5.5(5.2–5.8)	5.5(5.2–5.8)	0.232
Hs-CRP (mg/l)	1(1–2)	1(0–2)	0.0001
Uric acid (mg/dl)	5.7(4.8–6.8)	4.3(3.6–5.2)	0.0001
Total cholesterol (mg/dl)	182.9(160.1–208.4)	185.2(160.5–212.7)	0.014
Triglycerides (mg/dl)	116(77.9–189.5)	108.1(74.4–162.1)	0.0001
HDL-C (mg/dl)	50.7(42.5–60.7)	55.3(47.1–64.6)	0.0001
LDL-C (mg/dl)	110.6(88.9–133)	113.3(90.9–138.8)	0.0001
ApoA1(mg/dl)	1.07(0.92–1.27)	1.12(0.97–1.3)	0.0001
ApoB (mg/dl)	0.88(0.72–1.07)	0.88(0.71–1.07)	0.563
Apo B/Apo A1	0.81(0.63–1.03)	0.76(0.6–0.96)	0.0001
LPA (mg/dl)	7.2(3.5–15.3)	8.4(4.5–17.8)	0.0001
Sleep duration (hours)	8(7–8)	8(7–8)	0.053

BMI: body mass index; WC: waist circumference; FPG: fast plasma glucose; HbA1c: haemoglobin A1c; Hs-CRP: hs-C-reactive protein; HDL-C: high density lipoprotein cholesterol; LDL-C: low density lipoprotein cholesterol; ApoB: apolipoprotein B; ApoA1: apolipoprotein A1; LAP: lipid accumulation product

Tables 2 and 3 show the demographic and clinical characteristics of participants across sleep duration in men and women. As expected, short sleepers were older, long sleepers were more likely to live in rural regions, and optimal sleepers had the highest education level in both sexes. Among males, short sleep duration was associated with higher diastolic blood pressure, greater WC, and higher apoA1 and apoB compared with optimal and long sleepers (*P* < 0.05). Additionally, the optimal sleep duration group had the lowest hypertension compared with short and long sleepers. In women, we found that the percentage of current smokers and the percentages of women with elevated hypertension and diabetes, systolic and diastolic blood pressure, WC, FPG, HbA1c, uric acid, total cholesterol, triglycerides, LDL-C, apoB levels, and apoB/A1 ratios were significantly higher in short sleepers compared with optimal and long sleepers (*P* < 0.05), whereas drinking, BMI, HDL-C, and LPA levels were not different among groups (*P* > 0.05). Differences in hs-CRP and apoA1 levels were only significant between short and optimal sleepers in women (*P* < 0.05).

**Table 2:** Characteristics of the female participants across sleep duration

	5 to 6	7 to 8	9 to 10
N (%)	396(10.1)	2704(69)	818(20.9)
Age (years)	56.9(49.1–66.1)^*^	48.3(39.5–58)	49.3(38.9–59.7)^#^
Education < high school (%)	82.3^*^	76.6	87.2^*^
Rural region (%)	56.1^*^	67.3	72.9^*#^
Current smoking (%)	9.8^*^	2.9	3.9^#^
Drinking (%)	11.4	9.1	8.6
Medical history			
Hypertension (%)	21.5^*^	11.4	13.2^#^
Diabetes (%)	5.3^*^	1.8	2.4^#^
Systolic blood pressure (mmHg)	128(115–141)^*^	120(110–130)	120(109–131)^#^
Diastolic blood pressure (mmHg)	74(81–89)^*^	79(71-85)	79(70-83)^#^
BMI (kg/m2)	23.3(21.2–26)	23.1(21.1–25.6)	23.1(20.8–25.4)
WC (cm)	83(76.9–89)^*^	80(74–87.3)	80(74–87.8)^#^
FPG (mg/dl)	94.4(87.3–102.9)^*^	91.4(84.6–99.7)	90.4(84.1–19.5)^#^
HbA1c (%)	5.6(5.3–6)^*^	5.5(5.2–5.8)	5.5(5.2–5.8)^#^
Hs-CRP (mg/l)	1(1–3)^*^	1(0–2)	1(0–3)
Uric acid (mg/dl)	4.5(3.9–5.5)^*^	4.2(3.5–5.1)	4.2(3.5–5.2)^#^
Total cholesterol (mg/dl)	197.8(170.6–229.2)^*^	184.1(160.1–210.8)	181(157.8–210)^#^
Triglycerides (mg/dl)	119.1(77.9–185.1)^*^	107.2(73.5–158.5)	109(72.6–162.1)^#^
HDL-C (mg/dl)	54.9(46.4–64.9)	55.7(47.2–65)	55(46.8–63)
LDL-C (mg/dl)	124.1(96.8–150)^*^	112.5(90.9–137.3)	111(88.2–134.9)^#^
ApoA1(mg/dl)	1.16(0.98–1.36)	1.12(0.97–1.29)	1.11(0.97–1.29)
ApoB (mg/dl)	1(0.79–1.2)^*^	0.87(0.71–1.06)	0.85(0.7–1.03)^#^
ApoB/apoA1	0.84(0.64–1.04)	0.76(0.6–0.95)	0.74(0.6–0.93)^#^
LPA (mg/dl)	9.2(5–18.6)	8.4(4.4–17.9)	8.5(4.3–16.4)

^*^P < 0.05 compared with the reference 7 to 8 h; ^#^*P* < 0.05 compared with the reference 5 to 6 h; BMI: body mass index; WC: waist circumference; FPG: fast plasma glucose; HbA1c: heamoglobin A1c; Hs-CRP: hs-C-reactive protein; HDL-C: high density lipoprotein cholesterol; LDL-C: low density lipoprotein cholesterol; ApoB: apolipoprotein B; ApoA1: apolipoprotein A1; LAP: lipid accumulation product

**Table 3:** Characteristics of the male participants across sleep duration

	5 to 6	7 to 8	9 to 10
N (%)	303(9.2)	2362(68.2)	738(22.6)
Age (years)	54.6(45.1–63.6)^*^	49(39.1–58.6)	51.2(37.8–61.4^)#^
Education < high school (%)	72.3	69.3	77.8^*^
Rural region (%)	55^*^	69	73.1^#^
Current smoking (%)	59.4	56.1	55.2
Drinking (%)	64.8	62.5	57.3^*#^
Medical history			
Hypertension (%)	16.4^*^	10.2	14.6^*^
Diabetes (%)	4.4	3.2	3.4
Systolic blood pressure (mmHg)	122(115–138)	121(114–132)	121(112–135)
Diastolic blood pressure (mmHg)	81(77–90)	81(76–89)	80(73–88)^*#^
BMI (kg/m2)	23.2(21–25.8)	23.3(21.1–25.5)	23(20.7–25.4)^*^
WC (cm)	84.7(78–92)	84.5(77.4–91.3)	83.2(76–90.4)^*#^
FPG (mg/dl)	92.9(83.9–104.6)	92.5(85.1–102.1)	91.6(84.6–101.2)
HbA1c (%)	5.5(5.3–5.9)	5.5(5.2–5.8)	5.5(5.2–5.8)
Hs-CRP (mg/l)	1(1–3)	1(1–2)	1(1–2)
Uric acid (mg/dl)	5.7(4.7–6.7)	5.7(4.8–6.7)	5.7(4.8–6.6)
Total cholesterol (mg/dl)	181.8(160.1–213.1)	183.3(160.5–209.6)	180.6(158.1–204.8)
Triglycerides (mg/dl)	121.4(77.9–189.5)	116(77.9–192.4)	116.5(78–186.9)
HDL-C (mg/dl)	51.4(43.3–62.3)	50.3(42.1–61.1)	50.7(42.8–59.9)
LDL-C (mg/dl)	110.2(88.9–136.5)	110.6(89.3–133.8)	108.5(88.1–129.5)
ApoA1(mg/dl)	1.12(0.95–1.36)^*^	1.07(0.91–1.27)	1.06(0.92–1.24)^#^
ApoB (mg/dl)	0.9(0.74–1.12)	0.89(0.73–1.07)	0.85(0.71–1.03)^*#^
ApoB/apoA1	0.78(0.63–1.04)	0.82(0.63–1.04)	0.8(0.63–1)
LPA (mg/dl)	8.1(3.8–15.3)	6.9(3.4–15.1)	7.9(3.8–16)

^*^P < 0.05 compared with the reference 7 to 8 h; ^#^*P* < 0.05 compared with the reference 5 to 6 h; BMI: body mass index; WC: waist circumference; FPG: fast plasma glucose; HbA1c: hemoglobin A1c; Hs-CRP: hs-C-reactive protein; HDL-C: high density lipoprotein cholesterol; LDL-C: low density lipoprotein cholesterol; ApoB: apolipoprotein B; ApoA1: apolipoprotein A1; LAP: lipid accumulation product

**Fig. 1:**
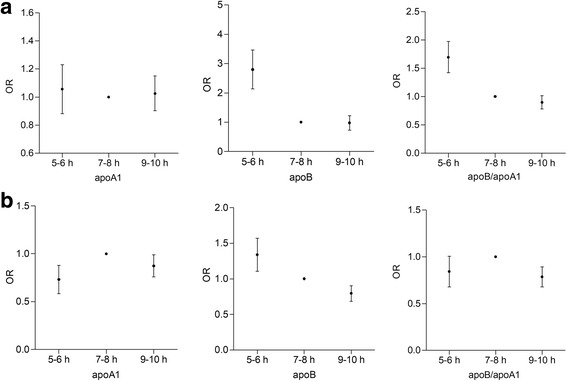
Crude odds ratio (OR) of decreased apoA1, elevated apoB and apoB/apoA1 ratio for sleep categories with the reference 7 to 8 h in women (**a**) and men (**b**)

Figure 1 shows the sex-specific crude association of sleep duration and apoA1, apoB, and the apoB/A1 ratio. The results showed that higher apoB levels and higher apoB/A1 ratio were positively associated with short sleep duration in women and were negatively related to long sleep duration in men compared with the reference 7 to 8 h (women OR 2.64, 95% CI 1.74–4.02, *P* < 0.0001; OR 1.68, 95% CI 1.3–2.18, *P* < 0.001; men OR 0.78, 95% CI 0.61–0.99, *P* = 0.046; OR 0.77, 95% CI 0.61–0.98, *P* = 0.03). Female participants with long sleep duration were more likely to have decreased apoB levels and apoB/apoA1 ratios compared to optimal sleep participants, though the difference was not significant (*P* > 0.05). We observed no association between apoA1 and sleep duration in either sex (*P* > 0.05).

However, when controlling for age and other cofounders, short sleep was no longer associated with the apoB/A1 ratio in women (Table 4 OR 1.25, 95% CI 0.94–1.65, *P* = 0.108). Interestingly, the association between short sleep and elevated apoB levels was still maintained after additional adjustment for potential confounders (OR 1.75, 95% CI 1.12–2.72, *P* = 0.013). In men, the negative association of long sleep with an elevated apoB/A1 ratio was increased with the inclusion of age, current smoking, drinking, less than high school education, urban region, medical history of hypertension and diabetes, systolic and diastolic blood pressure, FPG, HbA1c, urid acid, and hs-CRP in subsequent Models 1, 2 and 3 (OR 0.77, 95% CI 0.61–0.97, *P* = 0.029; OR 0.75, 95% CI 0.59–0.96, *P* = 0.021; OR 0.74, 95% CI 0.58–0.94, *P* = 0.015). However, the strength of the association was only marginally significant after further adding BMI and WC into model 4 (OR 0.78, 95% CI 0.6–0.99, *P* = 0.046). In addition, the adjusted association of decreased apoB levels and long sleep was not statistically significant across sleep duration groups (OR 0.84, 95% CI 0.65–1.1, *P* = 0.122).

**Table 4:** Adjusted odds ratios of decreased apoA1, elevated apoB and apoB/apoA1 ratio by sleep duration in each sex (with the reference 7 to 8 h)

	Model 1^a^				Model 2^b^		Model3^c^			Model 4^d^		
	ApoA1	ApoB	ApoB/A1	ApoA1	ApoB	ApoB/a1	ApoA1	ApoB	ApoB/A1	ApoA1	ApoB	ApoB/A1
Sleep duration(women)												
5 to 6	1.12(0.83–1.49)	1.83(1.189–2.82)	1.25(0.96–1.64)	1.09(0.812–1.47)	1.78(1.15–2.75)	1.21(0.92–1.59)	1.09(0.81–1.46)	1.71(1.1–2.65)	1.19(0.9–1.56)	1.123(0.84–1.51)	1.75(1.12–2.72)	1.25(0.92–1.65)
7 to 8	–	–	–	–	–	–	–	–	–	–	–	–
9 to 10	1.01(0.82–1.25)	0.84(0.53–1.33)	0.834(0.66–1.06)	1.0(0.81–1.25)	0.83(0.52–1.32)	0.83(0.66–1.05)	1.0(0.81–1.24)	0.86(0.54–1.38)	0.86(0.67–1.09)	1.02(0.82–1.27)	0.86(0.54–1.38)	0.87(0.69–1.11)
*P*	0.762	0.006	0.1	0.832	0.009	0.07	0.86	0.018	0.146	0.732	0.013	0.108
Sleep duration(men)												
5 to 6	0.78(0.54–1.12)	1.22(0.9–1.65)	0.79(0.56–1.12)	0.76(0.53–1.1)	1.19(0.88–1.62)	0.75(0.53–1.06)	0.77(0.53–1.11)	1.2(0.87–1.56)	0.75(0.53–1.06)	0.76(0.53–1.11)	1.22(0.89–1.67)	0.77(0.54–1.09)
7 to 8	–	–	–	–	–	–	–	–	–	–	–	–
9 to 10	0.86(0.68–1.08)	0.78(0.61–0.99)	0.77(0.61–0.97)	0.834(0.66–1.06)	0.8(0.63–1.02)	0.75(0.59–0.96)	0.82(0.65–1.04)	0.81(0.63–1.04)	0.74(0.58–0.94)	0.85(0.67–1.08)	0.84(0.65–1.07)	0.78(0.6–0.99)
*P*	0.22	0.039	0.029	0.153	0.073	0.021	0.124	0.092	0.015	0.182	0.122	0.046

^a^Model 1 was adjusted for age; ^b^Model 2 was adjusted for age, smoking status, alcohol consumption, education level, rural/urban, region and medical history of hypertension and diabetes. ^c^Model 3 was additionally adjusted for systolic and diastolic blood pressure, fast plasma glucose, HbA1c, uric acid, and hs-CRP. ^d^Model 4 was adjusted for all the variables in model 3 plus body mass index and waist circumference

Finally, we combined sex in the logistic regression analyses described above. A similar association between short sleep duration and elevated apoB levels was noted (OR 1.43, 95% CI 1.11–1.84); however, no significant association of sleep duration with serum apoA1 level or the apoB/apoA1 ratio was observed after adjustment for sex, age and other covariables.

## Discussion

This study took advantage of a representative sample of participants from the CHNS, and it demonstrated a significant increase in apoB levels among female participants with sleep deprivation and a decrease in the apoB/apoA1 ratio among male participants who slept longer. Short sleep duration was more strongly associated with apo levels (particularly apoB concentrations in women) than the other sleep duration groups. The current results extend upon the observations in previous studies suggesting that apoB levels and the apoB/apoA1 ratio are related to sleep duration and go further by providing additional information on the pathophysiological association of sleep duration with cardiometabolic disorders, as well as highlight the possibility of managing and preventing cardiometabolic diseases by controlling some of their risk factors through specific sleep interventions.

We have not been able to find previous studies exploring the association of sleep duration with apo profile. However, emerging evidence from several population-based studies have shown that short sleep duration may play critical roles in the pathogenesis of diabetes [25], obesity [26], and hypertension [27], all of which are primary risk factors for CHD. Observational studies have also implicated short sleep duration in the aetiology of atherosclerotic dyslipidaemia, such as high LDL-C, high triglyceride, and low HDL-C levels [15]. Similarly, our study reports that short sleep duration is positively correlated with high apoB concentrations. It is established that higher apoB levels are often correlated with an increased risk of developing CHD. Elevated levels of serum apoB and the apoB/apoA1 ratio, as well as a decreased apoA1 level, have been reported to be risk factors for CHD, independently of the traditional cardiovascular risk factors, and they could be superior predictive indicators of cardiovascular events [28, 29]. The high prevalence of dysregulation of these apo parameters in short sleep duration may predispose people to a high risk for CVD. Thus, these observations are important to understand the link between sleep duration and CHD.

Based on previous evidence, several mechanisms could mediate the link between short sleep duration and high apoB levels. Mechanisms such as a decrease in the serum leptin levels or an increase in the serum ghrelin levels due to sleep insufficiency may contribute to the correlation between short sleep duration and the dysregulation of apo variables. As reported in previous experimental studies, sleep restriction was associated with a reduced blood concentration of leptin which, in turn, increases the serum ghrelin levels, consequently increasing appetite and food intake [30]. The hormone leptin has a considerable impact on lipid metabolism. Evidence from an experimental study showed that a hepatocyte-specific loss of leptin signalling was associated with larger apoB-containing lipoproteins and elevated levels of very low-density lipoprotein (VLDL) triglycerides [31]. Results from a population-based cross-sectional study on the effect of leptin and adiponectin on cardiovascular disease (CVD) risk in Asian Indian adults, have shown that individuals with abnormal leptin levels had significantly higher apoB levels and apoB/apoA1 ratios, consequently having higher risk for CVD [32]. In addition, having a sleep deficit was hypothesized to increase the risk for unfavourable apo profile by promoting elevations in markers of inflammation. It was recently reported that sleep loss could lead to a chronic elevation of inflammatory markers levels, including interleukin-6, tumour necrosis factor-α, and hs-CRP [13, 33]. The inflammatory pathway may be involved in the biological progression responsible for the associations between sleep loss and abnormal apo variables.

Furthermore, we observed that long sleep duration is inversely with a high apoB/apoA1 ratio. In contrast to the short sleep-high apoB relationship, long sleep duration decreased the risk of abnormal apoB/A1 ratio. These findings may suggest that longer sleep may play an important role in CVD prevention. Regarding the associations between long sleep duration and metabolic/cardiovascular disorders, studies have reported inconsistent conclusions. King et al. have reported that longer measured sleep duration was negatively associated with the incidence of coronary artery calcification [34]. In a recent meta-analysis of prospective studies, however, no association was observed between long sleep duration and obesity risk [9]. These inconclusive results have not been clearly explained. We hypothesized that the insufficient adjustment for confounding factors, such as exercise habits, health behaviours, medical conditions, emotional distress, and socioeconomic factors in the literature, would partly explain the differences in long sleep duration and metabolic/cardiovascular disorder associations. Additionally, in previous studies, there are variations in the cut-off value of short or long sleep duration. Thus, the association of long sleep duration and apo variables needs to be considered with caution and confirmed in future studies.

It is also important to note that we did not observe a U-shaped association between sleep duration and apo variables. The monotonic trend of the association between sleep duration and apoB in women was found in the present study, though the magnitude in long sleep duration is not significant. Similar results were found by Gangwisch et al. [14]; a positive longitudinal association between short sleep duration and high cholesterol was observed among females, and a negative but not significant association between long sleep duration and hypercholesterolemia was noticed in males. Additionally, a community-based cross-sectional study has shown that short sleep duration increased the odds ratios of obesity in men, while long sleep duration decreased the risk of obesity in women [26]. In contrast, a recent meta-analysis has shown that the lowest risk of T2DM was observed among those who slept for 7 to 8 h, whereas both short and long sleep duration are associated with increased T2DM risk [20]. Furthermore, analyses of a data of 8860 participants from the Hordaland Health Study [35] showed a U-shaped association between sleep duration and BMI, where individuals who slept below 6 h and above 9 h had increased BMI compared with individuals who slept the reference sleep duration of 7 to 7.99 h. As we mentioned above, there is no consensus whether the link between long sleep and cardiometabolic diseases is causal or simply reflects confounding by other potential factors that disturb sleep quality. The underlying mechanisms still need to be further clarified.

The findings in the present study should be interpreted considering several limitations. First, because it is a cross-sectional study, the causal relationship between sleep duration and apo profile is undetermined. Further, it is also to be noted that the sleep duration was self-reported from a questionnaire and was not objectively measured. These findings should be confirmed in longitudinal studies using objective sleep measures. Though several studies have reported that both self-reported sleep duration and polysomnographic monitoring are reasonably accurate and highly correlated [36], other studies have suggested that self-reported sleep duration was longer than those directly measured [37].

## Conclusions

In conclusion, the present study indicates that short sleep duration could be a significant risk factor for elevated apoB levels in women, while long sleep duration is associated with decreased apoB/apoA1 levels in men. As sleep duration is a well-known modifiable risk factor, these findings could have significant clinical implications for the regulation of unfavourable apo profile and, consequently, for the prevention and treatment of CHD.

## Abbreviations

Apo: Apolipoprotein
apoB/apoA1: Apolipoprotein B/apolipoprotein A1
BMI: Body mass index
CHD: Coronary heart disease
CHNS: China Health and Nutrition Survey
CHOD-PAP: Cholesterol oxidase-phenol and aminophenazone
CVD: Cardiovascular disease
eGFR: Estimated glomerular filtration rate
FPG: Fast plasma glucose
GOD-PAP: Glucose oxidase-phenol and aminophenazone
GPO-PAP: Glycerol-3-phosphate oxidase-phenol and aminophenazone
HbA1c: Haemoglobin A1c
HDL-C: High-density lipoprotein cholesterol
hs-CRP: Hypersensitive C-reactive protein
IQR: Interquartile ranges
LDL-C: Low-density lipoprotein cholesterol
T2DM: Type 2 diabetes mellitus
VLDL: Very low-density lipoprotein
WC: Waist circumference
